# Comparison of two cell-free therapeutics derived from adipose tissue: small extracellular vesicles versus conditioned medium

**DOI:** 10.1186/s13287-022-02757-8

**Published:** 2022-03-03

**Authors:** Chuan He, Minjia Dai, Xiaojie Zhou, Jie Long, Weidong Tian, Mei Yu

**Affiliations:** 1 State Key Laboratory of Oral Disease and National Clinical Research Center for Oral Diseases and National Engineering Laboratory for Oral Regenerative Medicine, West China School of Stomatology, Sichuan University, Chengdu, China; 2 Engineering Research Center of Oral Translational Medicine, Ministry of Education, West China School of Stomatology, Sichuan University, Chengdu, China; 3 Department of Oral and Maxillofacial Surgery, West China Hospital of Stomatology, Sichuan University, Chengdu, China; 4 Department of Stomatology, The Second Affiliated Hospital of Soochow University, Suzhou, China

**Keywords:** Adipose tissue extract, Small extracellular vesicles, Adipogenesis, Angiogenesis, Regeneration

## Abstract

**Background:**

Cell-free therapy has been inspired as a promising approach to overcome the limitations of traditional stem cell therapy. However, the therapeutic effect between extracellular vesicles and conditioned medium with the same source had not been compared. Our previous studies have shown that both the conditioned medium of adipose tissue (adipose tissue extract, ATE) and its further purification product small extracellular vesicles (sEV-AT) contributed to adipose tissue regeneration. In this study, we aimed to compare the ATE and sEV-AT in composition, inductivity on cells and de novo adipose regenerative potential.

**Methods:**

The characteristics of sEV-AT and ATE were compared through protein and particle yield, particle size distribution and composition. The inductivity of sEV-AT and ATE on cells were compared through co-culture of sEV-AT or ATE with ASC, HUVEC and RAW264.7 in vitro. The capacity of promoting de novo adipogenesis was compared by implanting the silicone tube containing sEV-AT or ATE subcutaneously in vivo.

**Results:**

More particles and concentrated particle size distribution were detected in sEV-AT. In turn, more soluble factors and multiple peaks in particle size distribution were detected in ATE. In 1662 common proteins of sEV-AT and ATE, there were 984 (59.2%) proteins enriched twice more in sEV-AT than in ATE. With the prerequisite of equivalent protein concentration, sEV-AT outperformed ATE in promoting proliferation, migration and regeneration potential of cells those contributing adipose tissue regeneration in vitro*.* Furthermore, sEV-AT expedited the de novo adipose tissue regeneration and angiogenesis at the early stage than ATE in vivo, but sEV-AT and ATE group formed similar neoadipose tissue and new vessels at week 12.

**Conclusions:**

Our results provided a direct comparison between EV and conditioned medium as cell-free therapeutic strategy. Both sEV and ATE had specific biological signature to facilitate tissue repair. Considering the convenience of extraction and acceptable effect, ATE represented a feasible product of cell-free therapy, providing another option for different situations in clinical application. Furthermore, the complex contents of both sEV-AT and ATE should be studied comprehensively to avoid possible negative effects and to ensure sufficient safety for clinical applications.

## Introduction

Stem cells have been widely studied in tissue engineering and regenerative medicine for its variety of sources, abilities of self-renewing and potential of multidirectional differentiation. Benefited from these characteristics, stem cells have received much attention on their application in clinical therapies [1]. It has been traditionally assumed that the elemental therapeutic effect of stem cells was to achieve local and functional regeneration via their differentiation to strengthen or replace damaged tissues. However, tracing experiments of implanted stem cells in vivo indicated that mesenchymal stem cells (MSCs) might have too short life span to accomplish self-proliferation and differentiation into specific tissue cells [2, 3]. Besides, stem cell therapeutic application has also faced several challenges like immune rejection [4], tumor formation potential [5] and embolism [6–8].

Recently, there is growing evidence suggesting that it is bioactive factors (soluble factors, vesicles secreted from implanted stem cells, etc.) that matter during the treatment, other than stem cells themselves [9–11]. Therefore, cell-free therapy has been inspired as a promising approach to overcome the limitations of traditional stem cell therapy. Extracellular vesicles (EVs) and secretome have been attached much attention among cell-free therapies in tissue repair and regeneration. EV, especially exosomes, are loaded with ample selected cargoes, including proteins, lipids, nucleic acids, and glycoconjugates [12, 13], on which much concentration has been drawn. For instance, MSC-derived exosomes have been proven to be of help in bone and soft tissue regeneration [14, 15]. Sun et al. summarized the anti-inflammatory, anti-apoptotic, anti-fibrotic and pro-angiogenic effects of MSC-derived EV mainly working through exosomal microRNA [16]. In addition, Zhang et al. found that umbilical cord mesenchymal stem cells (UC-MSC) derived exosomes promoted cutaneous wound healing and angiogenesis in vivo [17]. Secretome, firstly used to name the collective term for all secreted proteins and secretory machinery of the bacteria by Tjalsma [18], has now been defined as the repertoire of molecules and biological factors secreted from cells into the extracellular space [9], containing various biologically active molecules such as growth factors, cytokines, chemokines, and extracellular vesicles. In most studies, conditioned medium is adopted to represent secretome, which has also been studied for many diseases and tissue repair [19, 20].

Although EV or conditioned medium with the same source may attain similar tissue repair and regeneration performance, there are still differences between EV and conditioned medium in contents and functions. Carceller et al. found that the anti-inflammatory effects of adipose derived stem cell conditioned medium (ASC-CM) on LPS-treated macrophages are not mediated by EV but the rest part of the ASC secretome [21]. Furthermore, Giannasi et al. discovered that it was ASC-CM other than ASC-EV that remarkably reduced TNFα-induced MMP activity of chondrocytes [22]. Consequently, it is necessary to compare EV and conditioned medium of the same source to clarify the discrepancy of their composition, efficiency in promoting regeneration, etc.

Our previous studies have demonstrated that both conditioned medium of adipose tissue (adipose tissue extract, ATE) [23] and small extracellular vesicles purified from ATE (sEV-AT) could promoted adipogenesis, angiogenesis and adipose tissue regeneration [24–26]. Nevertheless, neither ATE nor sEV-AT had been compared systematically. In this study, comparisons of ATE and sEV-AT on protein varieties, particle concentration and particle size distribution were made based on same concentration of proteins. Moreover, we compared the inductive effect of sEV-AT and ATE on adipose derived stem cell (ASC), human umbilical vein endothelial cell (HUVEC) and macrophages RAW264.7 in vitro and further compared their effect on neoadipose formation in vivo. The study is the first to compare sEV and conditioned medium derived from same origin quantitatively, paving the way for future application on cell-free therapy.

## Material and methods

### Preparation of sEV-AT and ATE

sEV-AT and ATE were prepared as described previously[23]. Briefly, 10 g inguinal fat pads of 4-week-old SD rats were cut into pieces in an aseptic operation, then cultured in Celstir Spinner Flask (Wheaton, USA) with 100 mL serum-free α-MEM (Hyclone, USA), 100 μg/mL penicillin and 100 μg/mL streptomycin (Solarbio, China) for 48 h. After removing cells and cell debris by centrifuging at 2,000 rpm for 10 min, the culture medium was further centrifuged at 5000*g*, 4 °C with Amicon® Ultra-15 Centrifugal Filter Units (3,000Mw cut-off membrane) (Millipore, USA) until concentrated to 20% of the original volume, termed as adipose tissue extract(ATE). Then ATE was further filtered through a 0.45 μm membrane to remove impurities, polymers and centrifuged at 5000 *g*, 4 °C with Amicon® Ultra-15 Centrifugal Filter Units (100,000Mw cut-off membrane) for 30 min to remove secrete factors. The retained part was mixed with 0.5 volume of Total Exosome Isolation™ reagent (Life Technologies, USA), incubated overnight at 4 °C and spun down for 1 h at 10,000 *g* at 4 °C. The pellet resuspended in PBS used as small extracellular vesicles derived from adipose tissue (sEV-AT) for further experiments.

### SDS-PAGE and Western blotting

30 μg sEV-AT and ATE were mixed with 4 × loading buffer (Solarbio, China) and boiled for 10 min, respectively. The proteins were separated on 12% SDS-PAGE gel electrophoresis (90 V, 120 min), then the protein bands were visualized by Coomassie Blue staining. For western blotting, the protein bands were transferred onto polyvinylidene fluoride membranes (Millipore, USA) (200 mA, 90 min) and incubated with specific antibodies (CD9, Zen, China 220642; aP2, Abcam, UK, ab108311; CAV-1, BBI, China, D161423) (1:1000) respectively followed by HRP-conjugated secondary antibody. Protein signals were detected via High sig ECL Western Blotting Substrate (Tanon, China), according to the manufacturer's protocol. Signals were visualized with a Chemiluminescence Apparatus (Tanon, China).

### Nanoparticle tracking analysis (NTA) and Dynamic light scattering (DLS)

sEV-AT and ATE were analyzed for particle concentration and size distribution using Zeta View (Particle Metrix, Germany) according to the manufacturer's instructions. To measure more extensive range of particle size distribution, sEV-AT and ATE samples were measured by DLS performed with Zetasizer Nano ZS90 (Malvern, UK). The performed analyses were repeated at least three times, and the mean values were reported.

### Cell culture

ASC were prepared and cultured as described previously [26]. Briefly, inguinal fat pads of 4-week-old SD rats were cut into pieces in an aseptic operation and removed visible blood vessels. Then the samples were treated with 0.5 mg/mL collagenase I (Sigma-Aldrich) solution at 37 °C for 30 min. After the dissociated tissue was centrifuged at 1200 rpm for 5 min, the fraction of cells was resuspended in DMEM (Gibco, USA) with 10% fetal bovine serum (FBS, Gibco, USA). The cells were trypsinized and passaged at a 1:3 ratio when they reached 80% confluence. Passage 3–5 ASC were used for the downstream experiments.

Human umbilical cords were obtained from the Department of Obstetrics of the West China Second University Hospital of Sichuan University with the signed informed consent from the parents as described previously [27].This experiment was conducted according to the World Medical Association Declaration of Helsinki and approved by Institutional Review Board of Sichuan University West China Hospital of Stomatology (WCHSIRB-D-2021-015). The collected umbilical cords were rinsed twice with PBS followed by filling with 0.2% collagenase I and incubating for 30 min in 37 °C and 5% CO_2_ for isolation of HUVEC. Then the cells were cultured in ECM (ScienCell, USA). The cells were trypsinized and passaged at a 1:3 ratio when they reached 80% confluence. Passage 3–5 HUVEC were used for the downstream experiments.

RAW264.7 were maintained in RPMI 1640 (Hyclone, USA) with 10% FBS. The cells were scraped and passaged at a 1:3 ratio when they reached 80% confluence.

### Cell proliferation

Cell proliferation was evaluated using Cell Counting Kit-8 (Keygen, China) following the manufacturer's instructions. ASC, HUVEC and RAW264.7 were seeded onto 96-well plates with the density of 2.5 × 10^3^, 500 and 10^4^ cells per well, respectively. Then the cells were treated with 50 μg/mL sEV-AT or ATE (0.1 mL per well). Growth curves were plotted according to the OD value which was measured at 450 nm using a spectrophotometer from day 1 to day 6 (*n* = 5).

### Scratch assay

Scratch assay was used to analyze the migration of cells. To ensure the same field during the image acquisition, parallel lines were marked on the reverse of plates. Then ASC, HUVEC and RAW264.7 were seeded onto 24-well plates with the density of 2 × 10^4^, 1 × 10^4^ and 2 × 10^5^ cells per well with basal culture medium, respectively. When cells contacted and formed a confluent monolayer, a straight line was scraped with a 200 μL pipet tip. After being washed with PBS to remove derbis, cells were co-cultured with 50 μg/mL sEV-AT or ATE (1 ml per well). The initial (0 h after scratching) and final (12 h after scratching) images were captured by phase-contrast microscope, and the scratch area was analyzed by image J.

### Induction of adipogenic differentiation

ASC at passage 4 were seeded in 12-well plates with the density of 4 × 10^4^ cells per well, then changed the culture medium (2 mL per well) as described below after 6 h: (1) group BLANK, α-MEM supplemented with 10% FBS; (2) group sEV-AT, α-MEM supplemented with 10% FBS, sEV-AT (50 μg/mL); (3) group ATE, α-MEM supplemented with 10% FBS, ATE (50 μg/ml); and (4) group adipogenesis (as a positive control), 1 mM DEX, 10 mM insulin, 200 mM indomethacin and 0.5 mM IBMX in α-MEM supplemented with 10% FBS. The medium was changed every 2 days. The cells were collected at day 5 or 10 for Real-Time PCR. And the adipogenic differentiation at day 10 was determined by Oil Red O (Sigma-Aldrich, USA) staining. The cells were fixed in 4% paraformaldehyde for 30 min followed by stained with 0.5% Oil O Red keep out of light for 30 min at room temperature. After capturing the phase-contrast images, Oil Red O in cells was extracted with 100% isopropanol for 15 min. The absorbance was measured at 510 nm using a spectrophotometer (Thermo, USA).

### Tube formation assay

HUVEC suspended with ECM at a density of 2 × 10^5^ cells/ml with or without sEV-AT or ATE(50 μg/mL) were seeded as 50 μL per well onto angiogenesis u-slide (Ibidi, Germany) pre-coated with 10 μL Matrigel (Corning, USA). After incubation for 4 h, phase-contrast images were captured by an inverted microscope. Total length, number of junctions and number of nodes were measured using the angiogenesis plug-in of Image J.

### Real-time PCR

RNA was isolated from collected cells using FastPure® Cell/Tissue Total RNA Isolation Kit (Vazyme, China) according to the manufacturer's protocol. For each sample, 500 ng of total RNA was reverse transcribed using PrimeScript RT reagent Kit with gDNA Eraser (Vazyme, China). For qPCR, the SYBR Green PCR master mix (Vazyme, China) was used. And qPCR was performed using the QuantStudio™ 6 Flex Real‐Time PCR System (Applied Biosystems). Gene expression was normalized to the endogenous control (GAPDH). The primer sequences are listed in Table 1.

**Table 1:** qRT-PCR primers

Gene names	Primer sequences
Rat-PPARγ2	
Forward	GCCCTTTGGTGACTTTATGGAG
Reverse	GCAGCAGGTTGTCTTGGATGT
Rat-aP2	
Forward	GTAGAAGGGGACTTGGTCTGTCAT
Reverse	ACTTTCCTGTCATCTGGGGTGA
Rat-Adiponectin	
Forward	GCCGTTCTCTTCACCTACGA
Reverse	CAGACTTGGTCTCCCACCTC
Human-VEGFA	
Forward	ATCGAGTACATCTTCAAGCCAT
Reverse	GTGAGGTTTGATCCGCATAATC
Human-CD31	
Forward	TCGTGGTCAACATAACAGAACT
Reverse	TTGAGTCTGTGACACAATCGTA
Human-Angiogenin	
Forward	ACCCTCACAGAGAAAACCTAAG
Reverse	GACGACGGAAAATTGACTGATC
Mouse-TNF-α	
Forward	ATGTCTCAGCCTCTTCTCATTC
Reverse	GCTTGTCACTCGAATTTTGAGA
Mouse-IL-1β	
Forward	ATCTCGCAGCAGCACATCAA
Reverse	ATGGGAACGTCACACACCAG
Mouse-TGFβ	
Forward	ATGGTGGACCGCAACAACGC
Reverse	GGCACTGCTTCCCGAATGTCTG
Mouse-IL-10	
Forward	AGCCTTATCGGAAATGATCCAGT
Reverse	GGCCTTGTAGACACCTTGGT
Mouse-CCL2	
Forward	CCACTCACCTGCTGCTACTCA
Reverse	TGGTGATCCTCTTGTAGCTCTCC
Mouse-CCL3	
Forward	GCAACCAAGTCTTCTCAGCG
Reverse	TGGAATCTTCCGGCTGTAGG
Mouse-CCL22	
Forward	ACCTCTGATGCAGGTCCCTAT
Reverse	TAAACGTGATGGCAGAGGGTG
Mouse-CXCL1	
Forward	CTGGGATTCACCTCAAGAACATC
Reverse	CAGGGTCAAGGCAAGCCTC
Mouse-CXCL2	
Forward	GAAGACCCTGCCAAGGGTTG
Reverse	AGGCAAACTTTTTGACCGCC
Mouse-CXCL12	
Forward	CGGTTCTTCGAGAGCCACAT
Reverse	GCCGTGCAACAATCTGAAGG
Rat-GAPDH	
Forward	TATGACTCTACCCACGGCAAG
Reverse	TACTCAGCACCAGCATCACC
Human-GAPDH	
Forward	CTTTGGTATCGGAAGGACTC
Reverse	GTAGAGGCAGGGATGATGTTCT
Mouse-GAPDH	
Forward	AAGAAGGTGGTGAAGCAGGCATC
Reverse	CGGCATCGAAGGTGGAAGAGTG

### Chemotaxis assay

Firstly, 3 × 10^5^ RAW264.7 cells were seeded in 24-well plates and treated with 50 μg/ml sEV-AT or ATE (500 μL culture medium per well) for 6 h. Then the culture medium containing 50 μg/mL sEV-AT or ATE was discarded and washed with PBS for 3 times followed by changing fresh culture medium without sEV-AT or ATE. These cells were termed as pre-treated RAW264.7. Then 1 × 10^4^ ASC, 1 × 10^4^ HUVEC or 1 × 10^5^ RAW264.7 were seeded onto the upper compartment of Transwell plate (Corning, USA) and moved into the 24-well plate of pre-treated RAW264.7. After co-cultured for 12 h, the cells having moved to the lower compartment were stained with crystal violet and counted to calculate the cells chemoattracted by pre-treated RAW264.7.

### Animal

8-week-old male nude mice (25–30 g, *n* = 20) were purchased from Dashuo experimental animal Co., Ltd. (Chengdu, China). The animals were housed at 22–26 °C with a 12 h light/dark cycle. All operations of animals were approved by the Institutional Review Board of Sichuan University West China Hospital of Stomatology (WCHSIRB-D-2016-143). With mice were under general anesthesia by 1% pentobarbital sodium (10 mL/kg, intraperitoneal injection), two silicone tubes (inner diameter 4.87 mm, height 5 mm) were subcutaneously implanted into the back of mice one on each side which contained 200 μL pre-cured Matrigel (Corning, USA) with 200 μg sEV-AT or ATE, respectively. Mice were sacrificed at 2, 4, 8, and 12 weeks respectively (*n* = 5 per timepoint) for analysis of contents of implanted tubes.

### Haematoxylin and eosin (HE) staining

The implanted tubes and their contents were fixed in 4% paraformaldehyde overnight at 4 °C. After removing the tubes, remaining tissues were dehydrated through a graded ethanol and paraffin embedded for hematoxylin and eosin (HE) staining to observe the morphology and count the capillary density.

### Bioinformatic analysis

Functional enrichment analysis (GO terms, biological pathways) of the ATE-specific proteins, and common proteins in sEV-AT and ATE was performed using FunRich (Version 3.1.3), which integrates heterogeneous genomic and proteomic resources. The corrected *p*-value (Benjamini–Hochberg method) was used to assess the statistical significance of the enrichment. The corrected *p*-value of the bioinformatic analysis results presented in this study were all less than 0.05.

### Statistical analysis

All statistical analyses were performed using GraphPad Prism 7 software. All data are presented as the means ± SEMs.

An unpaired two-tailed Student’s *t* test is used to determine significant differences between two groups. The data of more than two groups were analyzed with ordinary one-way ANOVA followed by Tukey’s multiple comparisons test, *p < 0.05, **p < 0.01, ***p < 0.001, ****p < 0.0001.

## Results

### Characterization of sEV-AT and ATE

**Fig. 1:**
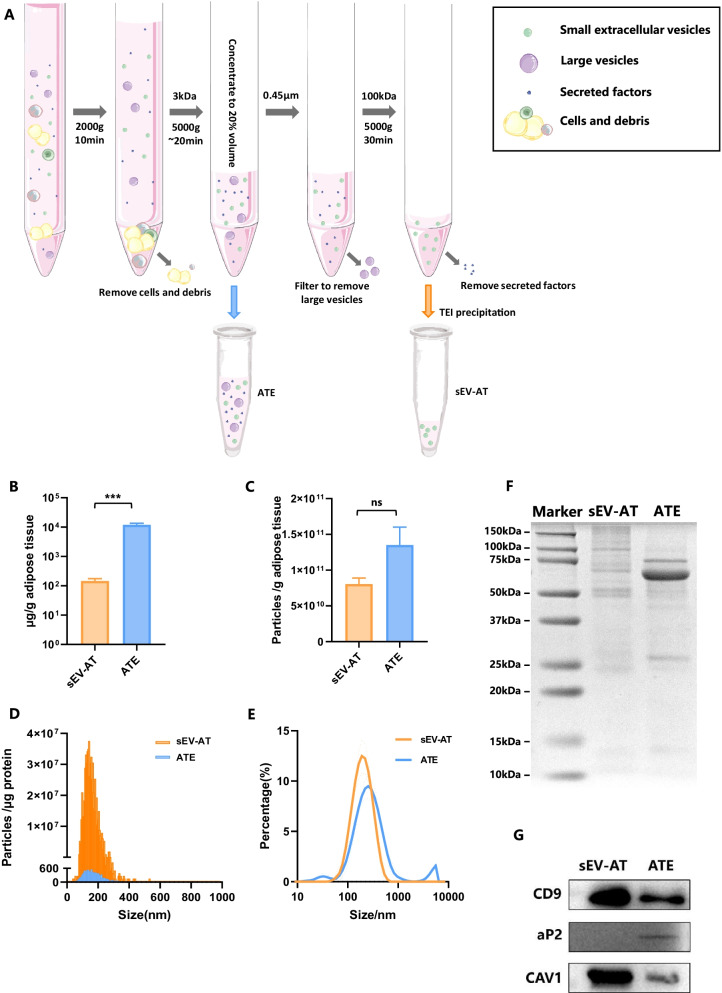
Characterization of sEV-AT and ATE. **A** Illustration of the difference on the preparation of sEV-AT and ATE. **B** Proteins of sEV-AT and ATE extracted from 1 g adipose tissue were quantified with BCA. The data were analyzed with unpaired two-tailed Student’s* t* test, ***p < 0.001. **C** Particles of sEV-AT and ATE extracted from 1 g adipose tissue were quantified with NTA. The data were analyzed with unpaired two-tailed Student’s *t* test. **D** Relative particles number and size distribution in 1 μg protein of sEV-AT and ATE. **E** Particle size distribution of sEV-AT and ATE tested with DLS, dashed lines illustrated the standard error of mean of three measurements. **F** Total proteins in sEV-AT and ATE were compared by SDS-PAGE and stained with Coomassie brilliant blue. **G** Proteins enriched in sEV-AT (CD9), ATE (aP2) and both(CAV-1) were validated with western blot

The preparation procedure of sEV-AT and ATE was presented in Fig. 1A. ATE was obtained by concentrating the adipose culture medium, and sEV-AT was obtained from ATE subsequently.

To compare the yield of sEV-AT and ATE from adipose, the quantity of proteins and particle number in sEV-AT and ATE from 1 g adipose tissue were measured. Given that sEV-AT was further purified from ATE, the amount of proteins and extracellular vesicles in ATE higher than that in sEV-AT. Specifically, the average protein yield of sEV-AT was 146 μg/g adipose tissue while that of ATE was 11890 μg/g adipose tissue, which was eight times higher than sEV-AT.

The particle concentration of sEV-AT and ATE was verified with NTA. From the results(Fig. 1B, C), we could tell that particle concentration of ATE was 1.3 × 10^11^ /g adipose tissue, reaching ~ 1.6 times higher than that of sEV-AT (8 × 10^10^ /g adipose tissue). What’s more, the particle concentration of sEV-AT was 5.5 × 10^8^ per microgram of proteins while that of ATE was 1.1 × 10^7^ /μg, which implied that sEV-AT contains more vesicles compared with ATE under the same amount of proteins, and ATE might work through soluble proteins (Fig. 1D). As for particle size, a broad size distribution was detected in ATE (from 30 to 5000 nm), while a single narrow peak showed up in sEV-AT (138 nm) (Fig. 1E).

The proteins in sEV-AT and ATE were separated by SDS-PAGE and visualized by Coomassie Blue Staining, and different patterns were exhibited between sEV-AT and ATE. Bands greater than 75 kD were observed more in sEV-AT than in ATE, suggesting discrepant protein enrichment (Fig. 1F). To further verify the characteristics of sEV-AT and ATE, expression of representative proteins were determined by western blot (Fig. 1G). CD9 was expressed significantly higher in sEV-AT than that in the ATE, considering CD9 is a specific membrane protein of exosomes [28]. On the other hand, aP2 was more abundant in ATE rather than in sEV-AT, which was consistent with the property of adipokine. Caveolin-1 (CAV-1) is at the same time an adipokine as well as presented in extracellular vesicles [29], thus it was observed both in sEV-AT and ATE. These results were in good agreement with previous research [30].

### Similarities and differences of proteins between sEV-AT and ATE

**Fig. 2:**
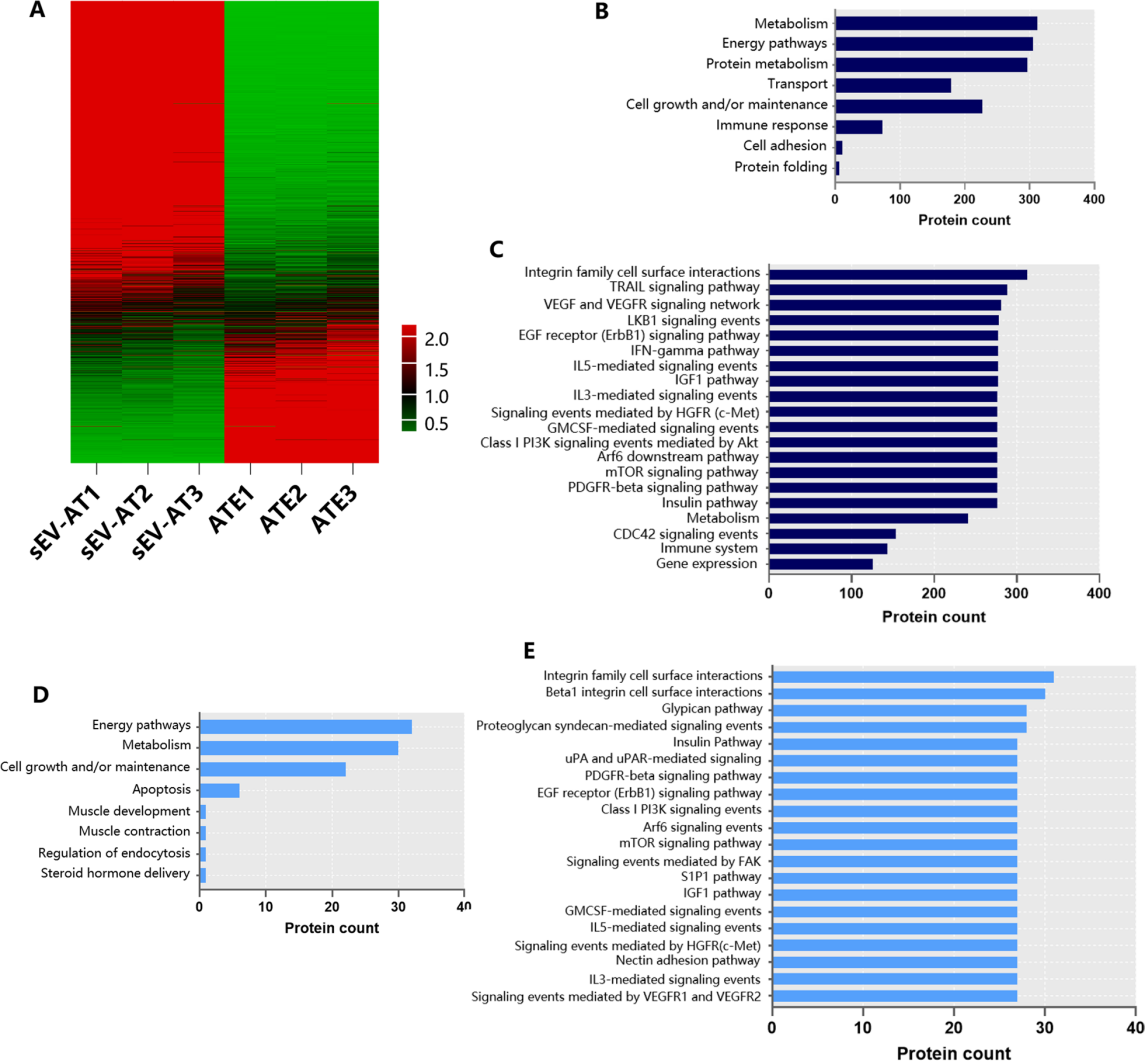
Similarities and differences of protein in sEV-AT and ATE. **A** Heat map showed the enrichment fold change of 1662 common proteins in sEV-AT compared with that in ATE. **B** GO-based categories clustering on biological processes of common proteins in sEV-AT and ATE. **C** Pathway enrichment analysis of common proteins in sEV-AT and ATE. **D** GO-based categories clustering on biological processes of ATE-specific proteins. **E** Pathway enrichment analysis of ATE-specific proteins

Label-free Quantitative Proteomic Analysis has been adopted to identify the proteins in sEV-AT and ATE [30]. Based on our previous study, we further analyzed common proteins in sEV-AT and ATE. 1662 common proteins were presented in sEV-AT and ATE, and their dgrees of enrichment in sEV-AT compared with that in ATE were showed in Fig. 2A. There were 984 (59.2%) proteins enriched more than twice in sEV-AT than in ATE, while there were only 366 (22.0%) proteins enriched more than twice in ATE. The expression levels of the rest 312 (18.8%) proteins showed no statistical difference, indicating that sEV-AT may have better performance in biological functions than ATE.

GO-based categories clustering (Fig. 2B) on biological processes and pathway enrichment analysis (Fig. 2C) demonstrated the substantial enrichment of these 1662 proteins in metabolism, energy pathway, protein metabolism, cells growth and/or maintenance, VEGF and VEGFR signaling network, Insulin, IGF-1 and PDGFR-β pathways, hinting that both sEV-AT and ATE might play a role in promoting cell migration and tissue regeneration.

Furthermore, GO-based categories clustering on biological processes of ATE-specific proteins (221 proteins) displayed 8 statistically significant results, among which only 3 kinds of enrichment were related to cell proliferation and adipose metabolism, including cell growth and/or maintenance, metabolism and energy pathways (Fig. 2D). Meanwhile, 945 proteins exclusive in sEV-AT showed 8 significant enrichment biological processes involved in cell proliferation and fat metabolism, which were signal transduction, cell communication, metabolism, energy pathways, protein metabolism, transport, cell growth and/or maintenance, lipid metabolism, cell proliferation, and protein folding [30]. As for the pathway enrichment analysis of ATE-specific proteins (Fig. 2E), most enriched pathways were consistent with those in common proteins (Fig. 2C). Besides, ATE-specific proteins were engaged in pathways related to growth factors and cytokine (VEGF and VEGFR, EGFR, IGF-1, PDGFR-β pathways, and IFN-γ, IL-3, IL-5, GM-CSF mediated signaling events) compared with results of sEV-AT specific proteins, from which it was suggested that soluble and secreted proteins like growth factors and cytokine were enriched more in ATE than in sEV-AT.

### sEV-AT and ATE promoted adipogenic differentiation of ASC

**Fig. 3:**
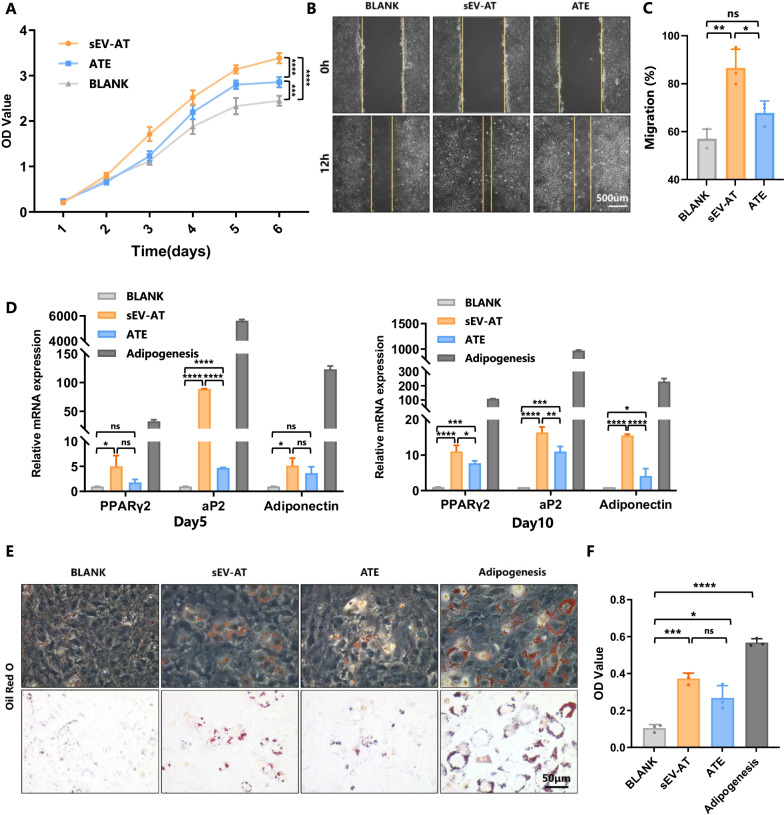
sEV-AT and ATE promoted adipogenic differentiation of ASC. **A** Growth curves showed the proliferation of ASC that co-cultured with sEV-AT or ATE. **B** Scratch assay showed the migration of ASC that co-cultured with sEV-AT or ATE. **C** Quantification of ASC migration. **D** Real-time PCR showed the expression of adipogenesis-related genes in ASC that co-cultured with sEV-AT or ATE at day 5 and day 10. **E** Oil O Red staining showed the adipogenesis of ASC that co-cultured with sEV-AT or ATE. **F** Quantification of Oil O Red staining. Data were represented as mean ± SEM. *n* = 3. *p < 0.05, **p < 0.01, ***p < 0.001, ****p < 0.0001

In view of the essential role of ASC in soft tissue engineering and regenerative medicine [31], we compared the effects of sEV-AT and ATE on the biological behavior of ASC. Both sEV-AT and ATE could promote the proliferation and migration of ASC, while sEV-AT had more impact than ATE. The ability to induce adipogenic differentiation of ASC was compared between sEV-AT and ATE (Fig. 3A–C). The expression of adipogenic-related genes examined by Real-Time PCR showed that sEV-AT had a more extraordinary ability to boost their expression. Adipogenesis induction medium was used as a positive control (Fig. 3D). After 10 days of co-culture with sEV-AT or ATE, Oil O staining was conducted to observe the lipid droplet formation. Though all the cells treated with either sEV-AT or ATE formed red lipid droplets around the nucleus, lipid droplets more in quantity and bigger in size were distinguished in the cells treated with sEV-AT than in ATE group (Fig. 3E), with which the OD value of eluted Oil O red was consistent with the staining result (Fig. 3F). Conclusively, sEV-AT had a stronger ability to induce adipogenic differentiation of ASC.

### sEV-AT and ATE promoted angiogenesis of HUVEC

**Fig. 4:**
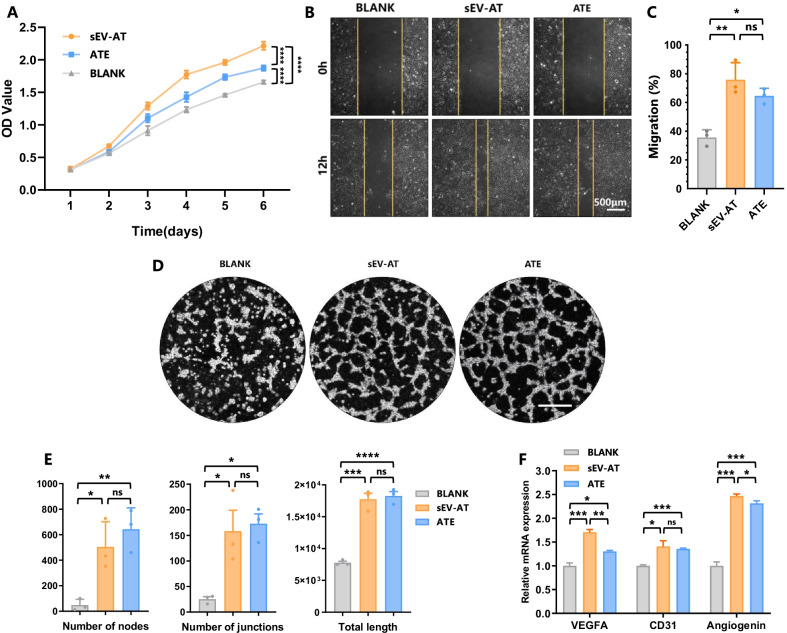
sEV-AT and ATE promoted angiogenesis of HUVEC. **A** Growth curves showed the proliferation of HUVEC that co-cultured with sEV-AT or ATE. **B** Scratch test showed the migration of HUVEC that co-cultured with sEV-AT or ATE. **C** Quantification of HUVEC migration. **D** Tube formation assay showed the angiogenesis of HUVEC that co-cultured with sEV-AT or ATE. **E** Quantification of angiogenesis with number of nodes, number of junctions and total length. **F** Real-time PCR showed the expression of angiogenesis-related genes in HUVEC that co-cultured with sEV-AT or ATE. Data were represented as mean ± SEM. *n* = 3. *p < 0.05, **p < 0.01, ***p < 0.001, ****p < 0.0001

Vascularization is necessary for the nourishment of regenerative tissue [32]. Hence, we compared the angiogenic inductivity between sEV-AT and ATE, including proliferation, migration, tube formation and expression level of angiogenic-related genes in HUVEC. It turned out that sEV-AT had a stronger impact on promoting HUVEC proliferation and migration (Fig. 4A–C), similar to the results of sEV-AT and ATE on ASC. In tube formation assay, sEV-AT and ATE group formed more tube-like structures than the blank group, there was no statistically significant difference in total length, the number of junctions or total nodes between the sEV-AT and ATE group (Fig. 4D, E). Additionally, the gene expression level of VEGFA, CD31 and Angiogenin were up-regulated both in sEV-AT and ATE group (Fig. 4F). These results indicated that both sEV-AT and ATE facilitated angiogenesis in vitro.

### sEV-AT significantly promoted the chemoattraction ability of RAW264.7 compared with ATE

**Fig. 5:**
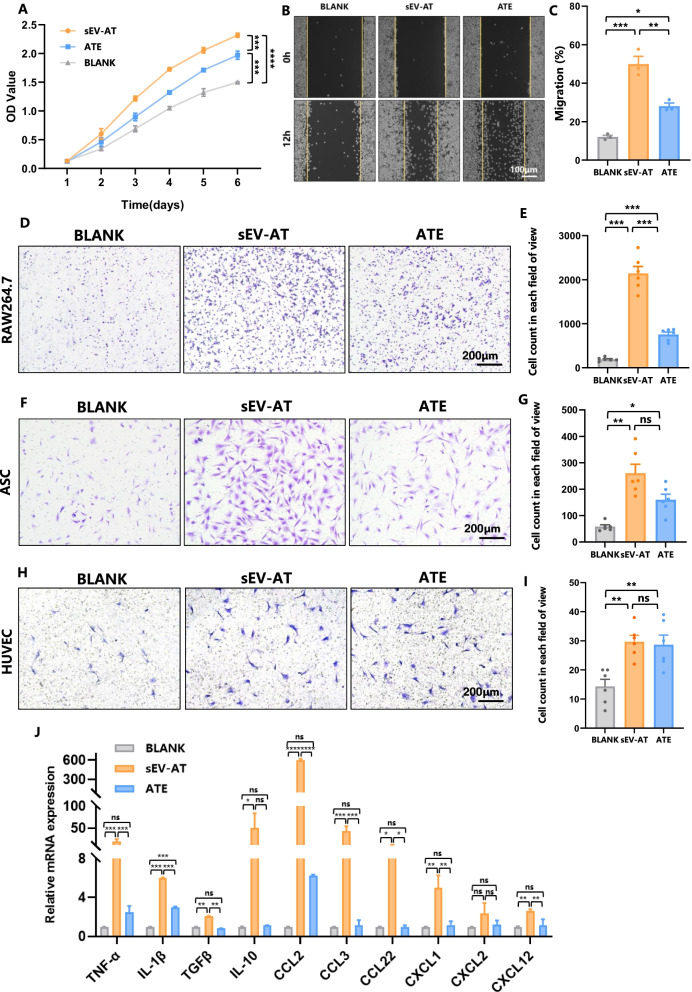
sEV-AT and ATE promoted the chemoattraction ability of RAW264.7. **A** Growth curves showed the proliferation of macrophages that co-cultured with sEV-AT or ATE. **B** Scratch test showed the migration of macrophages that co-cultured with sEV-AT or ATE. **C** Quantification of RAW264.7 migration. **D**, **E** chemoattraction of sEV-AT or ATE treated RAW264.7 on RAW 264.7 and the relative quantification. **F**, **G** chemoattraction of sEV-AT or ATE treated RAW264.7 on ASCs and the relative quantification. **H**, **I** chemoattraction of sEV-AT or ATE treated RAW264.7 on HUVECs and the relative quantification. **J** Real-time PCR showed the expression of cytokine in RAW 264.7 co-cultured with sEV-AT or ATE. Data were represented as mean ± SEM. *n* = 3. *p < 0.05, **p < 0.01, ***p < 0.001, ****p < 0.0001

Activated monocytes/macrophages are of great importance to mediate subsequent healing stages [33], tissue repair and angiogenesis in adult tissues by taking up foreign particles and expressing many cytokines, chemokines, and growth factors [34]. Therefore, the effect of sEV-AT and ATE on RAW264.7 were evaluated in vitro. Both sEV-AT and ATE could promote the proliferation and migration of RAW264.7, while sEV-AT demonstrated stronger impact than ATE (Fig. 5A–C). We next tested the chemoattraction ability of RAW264.7 treated with either sEV-AT or ATE. From Fig. 5D-I, it was indicated that the number of RAW264.7, ASC and HUVEC cells crossing the microposous membrane of Transwell all considerably increased in sEV-AT and ATE pre-treated group, clarifying that both sEV-AT and ATE promoted the chemoattraction ability of RAW264.7. To be more specific, compared with ATE pre-treated group, the chemoattraction ability of sEV-AT pre-treated RAW264.7 was 2.8 times higher as for RAW264.7 (Fig. 5D, E), 1.6 times higher as for ASC (Fig. 5F, G), and there was no significant difference between both group as for HUVEC (Fig. 5H-I). Afterwards, the expression of several cytokine genes in sEV-AT or ATE treated RAW264.7 were examined. The treatment of sEV-AT extensively increased the expression of TNF-α, IL-β, TGFβ, IL-10, CCL2, CCL3, CCL22, CXCL1 and CXCL12 in RAW264.7 compared with ATE (Fig. 5J). To sum up, RAW264.7 treated with both sEV-AT and ATE could be activated to express more cytokines, which promoted the migration of RAW264.7, ASC and HUVEC.

### sEV-AT accelerated de novo adipogenesis compared with ATE

**Fig. 6:**
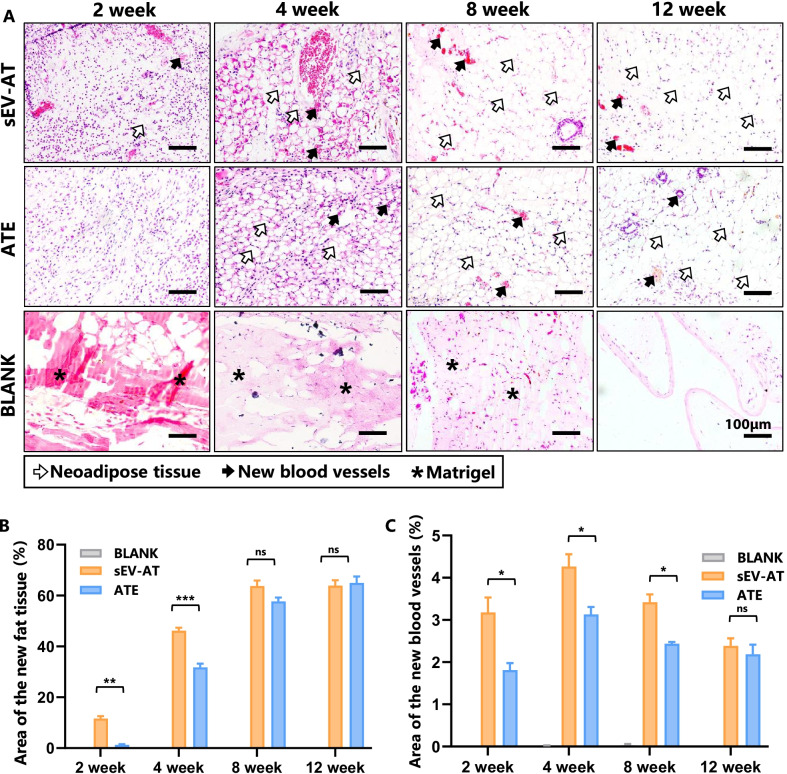
sEV-AT accelerated de novo adipogenesis. **A** H&E staining showed the progress of de novo adipogenesis prompted by sEV-AT and ATE. The hollow arrows represented the neoadipose tissue. The solid black arrows represented the new blood vessels, the asterisk represented the Matrigel. **B**, **C** The area percentage of the neoadipose tissue (**B**) and new blood vessels (**C**) in sEV-AT or ATE groups. Data were represented as mean ± SEM. *n* = 3. *p < 0.05, **p < 0.01, ***p < 0.001, ****p < 0.0001

The effect of sEV-AT and ATE on adipose regeneration was investigated in vivo by implanting a silicone tube that contained sEV-AT or ATE subcutaneously (Fig. 6A). After 2 weeks of implantation, considerable cell infiltration and some scattered immature adipocytes were seen in the implanted tube of sEV-AT group compared with the ATE group. At week 4, the clustered neoadipose tissue appeared in all groups except the BLANK group, while there were larger blood vessel and adipose areas in sEV-AT group than in ATE group. At week 12, the amount of mature adipose tissues and blood vessels showed no significant difference between sEV-AT and ATE group. In contrast, after Matrigel being absorbed, there were only fibrous capsules were observed in the BLANK group. The area changes of neoadipose tissues (Fig. 6B) and blood vessels (Fig. 6C) with the development of time were evaluated, respectively. These data indicated that both sEV-AT and ATE could induce adipose regeneration and eventual mature adipose tissue formation, while sEV-AT accelerated de novo adipogenesis process.

## Discussion

Our previous studies explored that sEV could be isolated from adipose tissue for further therapeutic and regeneration application. It was demonstrated that sEV-AT could stimulate the adipogenesis of ASC, promote the angiogenesis of HUVEC, thus could be used as a cell-free therapeutic approach for adipose tissue regeneration [25]. sEV-AT also contributed to bone and soft tissue regeneration in the rat model of bisphosphonate-related osteonecrosis of the jaw [27]. In addition, due to the complex composition of adipose tissue-derived EVs, they may provide many opportunities to modulate the metabolism of distant tissues through the delivery of EVs, for instance, their regulation on endothelial cell vascularization via HIF-1α, Akt, ERK, and SDF-1 [35]. In the meantime, adipose tissue extract (ATE), the secretome of adipose tissue, has also been studied. Early research in 2007 showed that ATE enhanced the expression of epithelial cell marker cytokeratin and fibroblast marker vimentin in the culture of rat skin in vitro, and the usage of adipose tissue paste stimulated PCNA expression and new blood vessel formation in mini pig wound healing model [36]. Sarkanen et al. tested the capability of ATE in inducing angiogenesis and adipogenesis in vitro [37]. Lo'pez et al. suggested that as for wound healing, ATE provided more optimal growth factors than platelet rich plasma (PRP) and promoted the proliferation and migration of various cells [38]. Lu et al. have observed the effect of ATE on adipose tissue regeneration in an engineering chamber model, in which regenerated adipose tissue of ATE group exhibited more considerable volume, better morphology and structure, a thinner capsule, and more vessels compared with control group [39]. Cai et al. revealed that ATE (named as fat extract in their research) isolated from human fat contained proangiogenic growth factors that promoted proliferation, migration, and tube formation in HUVEC in vitro, while the treatment of ATE increased capillary density of skin flap, thereby reducing flap necrosis in a rat model [40].

On the basis that both sEV-AT and ATE could promote adipose tissue regeneration, we compared them with the prerequisite of equivalent protein concentration. Although sEV-AT can be quantified by particle number, it was hard to quantified ATE in the same way, considering that ATE contains much less vesicles but more soluble factors than sEV-AT. Accordingly, quantification of protein concentration was used in this study, and the choice of concentration was based on the optimal dose of our previous studies [25]. From the aspect of isolation method, sEV-AT is a product of ATE with further condensation and enrichment, but this process removes some small molecules at the same time. Consequently, the proteomic comparison reflected that ATE contained more soluble molecules rather than vesicles, while sEV-AT contained more particles.

Though both sEV-AT and ATE could promote proliferation and migration of ASC and HUVEC, adipogenesis of ASC and angiogenesis of HUVEC, which was consistent with previous studies [23, 25, 26, 38], sEV-AT performed better in most aspects. The ability to recruit host cells to the regeneration site is an essential step in the cell-free regeneration process, and sEV-AT enabled this process to occur more quickly and strongly, which promoted adipose tissue regeneration more rapidly. Combined with the proteomic enrichment analysis, in spite of that there were 1662 shared proteins between sEV-AT and ATE, most of them were highly expressed in sEV-AT. Besides, the biological processes enriched in those shared proteins were more like those enriched in proteins exclusive in sEV-AT, indicating sEV-AT was an important part of the integrated secretome under the experimental condition of equal protein concentration.

After RAW264.7 was pretreated by sEV-AT and ATE, the expression of cytokine genes was significantly up-regulated, especially CCL2 (MCP-1), CCL3 (MIP-1) and TNF-$\alpha$. The release of these cytokines could further attract more host cells, including immune cells, stem cells and endothelial cells, to the regenerating site, which was then followed by repair and regeneration process [34]. In other words, it was advantageous that sEV-AT treatment raised pro-inflammatory cytokines release, because repair and regeneration are tightly linked to initial inflammation. Meanwhile, it was also important to control temporal coordination of inflammation resolution with other ongoing cellular processes [41], suggesting that sEV-AT and ATE treatment should be controlled within a proper range (concentration, frequency, etc.).

Although sEV-AT could accelerate adipose regeneration compared to ATE, ATE is also of importance to some extent in this process. As the adipose tissue conditioned medium, ATE contains various serum proteins, growth factors, hormones, cytokines, ECM proteins and proteases, and these secreted factors play pivotal roles in many biological activities [9]. Sarkanen et al. tested 120 growth factors and cytokines of human ATE by cytokine array, from which it could be told that angiogenin, leptin, IL-6, MIP-1a, IGF, bFGF, VEGF were highly expressed in ATE after being incubated in DMEM for 24 h [37]. In addition, the evidence in Sarkanen's study turned out that the adipogenic potential of ATE was dose-dependent. Given that the concentration of ATE used in our present study was at most 50 μg/mL, while the initial protein concentration of ATE we obtained was about 5–7 mg/mL, we proposed that ATE might have a better performance with an increase of its working concentration. Another possible reason for ATE underperformed sEV-AT is that the complex contents of ATE might bring unintended effect to cells. When compared with senescence-associated secretory phenotype (SASP) listed in Coppé J.P's review [42], IGFBP3, IGFBP5, MMP3, MMP10 belonging to SASP were found in proteins of ATE. These factors might affect ATE-treated cells and induce cell senescence, thereby attenuating the effect of ATE promoting cell proliferation and migration. Moreover, these factors could also assist senescent cells modify tissue microenvironment, which might be the reason why ATE had similar effect with sEV-AT on promoting de novo adipogenesis in vivo.

EV-based therapies have so far been used toward regeneration of various diseases and conditions. sEV-AT also exerted a more significant regenerative effect in this study. However, the preparation of pure small extracellular vesicles is inefficient, time/cost-intensive [43], lack of standard protocol, and the long-term preservation is inconvenient yet [44, 45]. Contrarily, the secretome with comparable effect in this study could be obtained relatively quicker and easier, promising it is a facile practical application in the future.

## Conclusion

Collectively, in this study, we initially analyzed similarities and differences of proteins in sEV-AT and ATE by bioinformatics. Based on the known facts that both sEV-AT and ATE work in promoting regeneration, we compared the small extracellular vesicles and conditioned medium derived from adipose tissue in their composition, capability of inducing cells and de novo adipose regeneration potential for the first time. Although sEV-AT surpassed on promoting cell proliferation in vitro and adipose tissue regeneration in vivo with the prerequisite of equivalent protein concentration, ATE had the advantage of easy availability and adequate performance. Regarding the factors mentioned above, ATE represented a feasible product of cell-free therapy, providing another option for different situations in clinical application. Furthermore, the complex contents of both sEV-AT and ATE should be studied comprehensively to avoid possible negative effects and to ensure sufficient safety for clinical applications.

## Data Availability

The datasets used and/or analyzed during the current study are available from the corresponding author on reasonable request.

## Abbreviations

ATE: Adipose tissue extract
sEV-AT: Small extracellular vesicles derived from adipose tissue
MSC: Mesenchymal stem cells
EV: Extracellular vesicle
UC-MSC: Umbilical cord mesenchymal stem cells
ASC-CM: Adipose derived stem cell conditioned medium
ASC: Adipose derived stem cell
HUVEC: Human umbilical vein endothelial cell
NTA: Nanoparticle tracking analysis
DLS: Dynamic light scattering
HE: Haematoxylin and eosin
CAV-1: Caveolin-1
PRP: Platelet rich plasma
